# Adsorption kinetics of Rhodamine-B on used black tea leaves

**DOI:** 10.1186/1735-2746-9-2

**Published:** 2012-08-02

**Authors:** Mohammad Abul Hossain, Md Shah Alam

**Affiliations:** 1 Department of Chemistry, University of Dhaka, Dhaka, 1000, Bangladesh

**Keywords:** Adsorption kinetics, Rhodamine B, Used black tea leaves, Adsorption enthalpy

## Abstract

Rhodamine B (Rh-B) is one of the most common pollutants in the effluents of textile industries effluents in developing countries. This study was carried out to evaluate the applicability of used black tea leaves (UBTL) for the adsorptive removal of Rh-B from aqueous system by investigating the adsorption kinetics in batch process. The effects of concentration and temperature on adsorption kinetics were examined. First-, second- and pseudo-second order kinetic equations were used to investigate the adsorption mechanism. The adsorption of Rh-B on UBTL followed pseudo-second order kinetics. The equilibrium amount adsorbed and the equilibrium concentration were calculated from pseudo-second-order kinetic plots for different initial concentrations of Rh-B to construct the adsorption isotherm. The adsorption isotherm was well expressed by Langmuir equation. The maximum adsorption capacity of UBTL to Rh-B was found to be 53.2 mg/g at pH = 2.0. The equilibrium amount adsorbed, calculated from pseudo-second-order kinetic plots, increased with temperature increase. The positive value of enthalpy of adsorption, ΔH_ads_ = 31.22 kJ/mol, suggested that the adsorption of Rh-B on UBTL at pH = 2.0 is an endothermic process.

## Background

Synthetic dyes are mostly used in industries such as textile, leather, paper and plastics to color their final products [1]. The effluents of textile, dying and finishing factories are colored because of the presence of dyes. Dyes may undergo chemical as well as biological change in aquatic medium and disturb the aquatic ecosystem [2].

Many types of dyes are not easily biodegradable under aerobic conditions. Some of them are considered carcinogenic. They can travel long distance in the surface water and cause special environmental concern. Therefore, to save human life and also aquatic living organisms, dyes from the wastewater of these factories must be removed before discharging into water-bodies.

Different methods are available for the removal of dye from wastewater including coagulation [3], chemical reaction [4], photo-degradation [5], bio-degradation [6,7], ultrasound-degradation [8] and reverse osmosis [9]. Among the above methods adsorption is considered to be easy and economic for the removal of dyes from aqueous systems. The successful application of adsorption demands the provision of cheap, easily available and abundant adsorbents with known kinetic parameters and adsorption characteristics.

Activated carbon has been considered as a standard adsorbent for the removal of different types of dyes; but the use of activated carbon is an expensive process due to its high cost and difficulties in the regeneration of adsorbed activated carbon. Therefore, the production of a low-cost alternative has become a focus to researchers for the last few years. Recently, used black tea leaves (UBTL) are considered in the region due to their high adsorption capacity and that the recovery of adsorbate from adsorbed UBTL is very easy [10].

Rhodamine B (Rh-B) was selected as a common basic dye in textile industries effluent, for its removal study. Very recently, equilibrium adsorption of Rh-B on UBTL was reported [11]. But the adsorption kinetics is an important physicochemical tool to evaluate the basic qualities and the proper use of an adsorbent. Therefore, the present study was carried out to investigate the kinetics of adsorption of Rh-B on UBTL under various experimental conditions.

## Materials and methods

### Adsorbent

**Figure 1:**
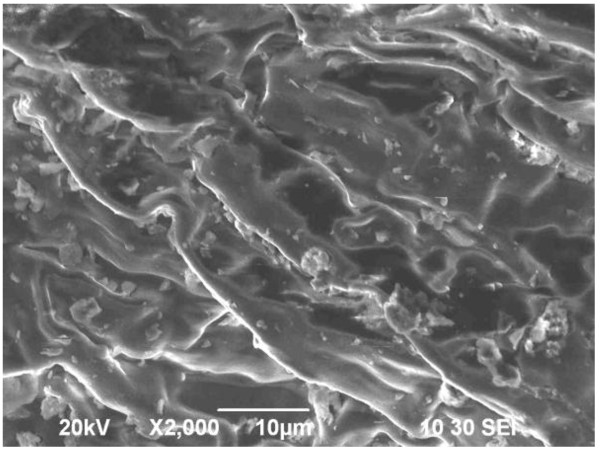
SEM micrograph of prepared used black tea leaves (UBTL).

Used black tea leaves (UBTL) were prepared from commercial fresh black tea leaves, collected from Dhaka City in Bangladesh, by boiling with distilled water for 8 h. After extraction of tea liquor, leaves were dried at room temperature and dried in oven at 105°C for 10 hours. Dried leaves were sieved through the sieves of mesh sizes of 0.30 and 0.42 mm and were stored in air-tight bottles for adsorption experiments. The nature of prepared UBTL surface was investigated by Scanning Electron Microscope (SEM) (JSM-6490LA, JEOL, Japan) as shown in Figure 1.

### Analysis of adsorbate

All chemicals used in the study were analytical grade. Commercial grade Rhodamine B (Rh-B) was collected from local market. A stock solution of 1000 mg/L was prepared by dissolving required amount of Rh-B in distilled water. For calibration curve, a series of different concentrated Rh-B solutions were prepared by required dilution of stock solution. The pH of each solution was adjusted at 2 using either 0.1 mol/L HCl or 0.1 mol/L NaOH, whichever was necessary. A UV-visible spectrometer (UV–vis 160A, Shimardzu, Japan) was used to measure the absorbance at pre-determined λ_max_ = 557 nm for each solution of different concentrations at pH = 2.0. A calibration curve was obtained from the plot of measured absorbance vs respective concentrations.

### Kinetic experiments

Adsorption kinetic experiments in batch process were carried out in a series of reagent bottles at a constant temperature, by suspending 0.1 g of the UBTL in 25 mL of constant concentrated Rh-B solution at fixed pH = 2.0, selected by preliminary experiments [12]. The bottles were placed in a thermostatic mechanical shaker (HAAKE SWB20, Fissions Ltd., Germany) at 30°C and were shaked continuously for different contact times. Reagent bottles were successively withdrawn from the shaker after definite interval time. Then the suspended UBTL was separated from solutions, the pH of the supernatant was adjusted at 2.0 and the absorbance was measured using UV–vis spectrometer at λ_max_ = 557 nm to determine the residual concentration of Rh-B. The initial concentration of Rh-B solution was also determined using the same analytical procedure. Similar experiments were performed for different initial concentrations of Rh-B, using same conditions of other parameters.

### Effect of temperature

To determine the effect of temperature on the adsorption kinetics, kinetic experiments were performed at three different temperatures using initial concentration of about 100 mg/L of Rh-B at pH = 2.0, keeping other parameters constant. The change of concentrations of Rh-B with time for different temperatures was estimated.

## Results

### Analysis of Rhodamine B

The concentration of Rh-B in solution was determined by UV-visible spectrometric method. From the verification of Beer-Lambert law in the plot of absorbance *vs* concentration, the calibration limit and the molar absorption coefficient of Rh-B at pH = 2 were determined which were 0.6 to 7.0 mg/L and 2.433 × 10^6^ L/mol·cm, respectively.

### Adsorption kinetics

**Figure 2:**
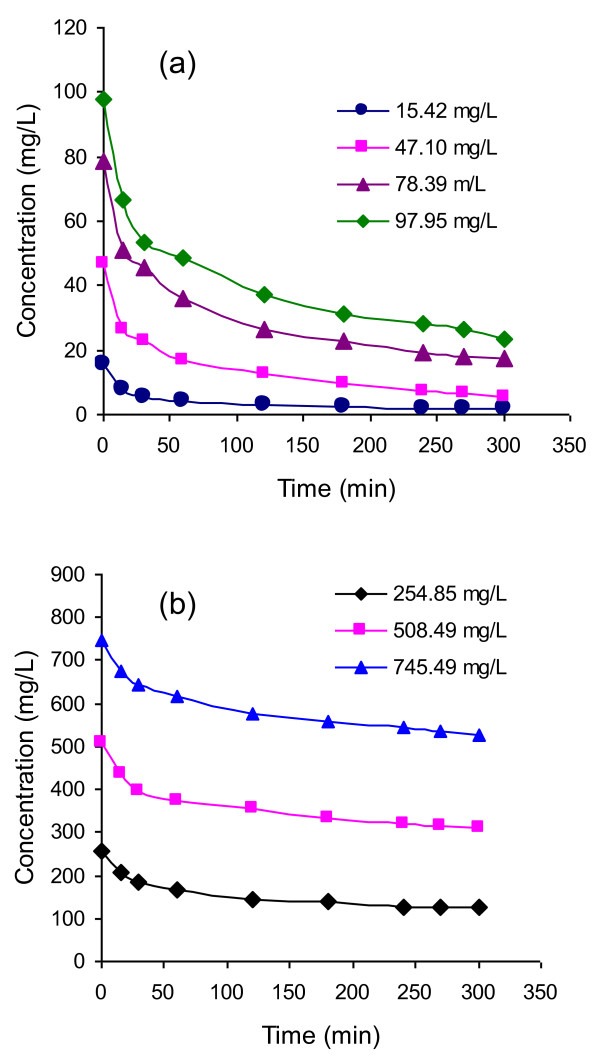
(a and b): Variation of the concentration of Rh-B with time for different initial concentrations at pH = 2.0 and 33 ± 0.5°C.

The change of concentrations of Rh-B solutions with contact time for different initial concentrations is presented in Figure 2. Figures 2a and 2b show that the concentration of Rh-B at various initial concentrations gradually decreased with time. As the time passed, the Rh-B accumulated on the surfaces of UBTL, so the concentration of Rh-B in solution decreased. The variation of Rh-B concentration with time was characterized by using different kinetic equations as follows:

### First-order kinetics

**Figure 3:**
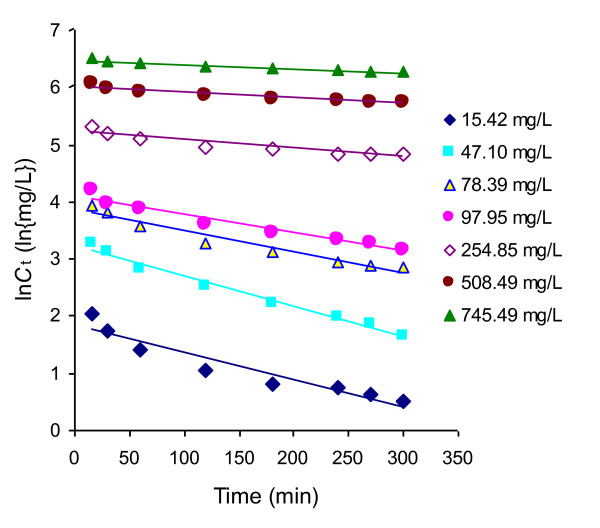
First order kinetic plots of the adsorption of Rh-B on UBTL at pH = 2.0 and 33 ± 0.5°C for different initial concentrations.

Several studies have been reported [13-15] about the applicability of simple first-order kinetic equation (1) to describe the adsorption of solid- liquid system:

(1) $$\text{ln}C_{\text{t}}=-k_{1}t+\text{ln}C_{\text{o}}$$

where, *C*_o_ is the initial concentration of adsorbate (mg/L), *C*_t_ is the concentration of adsorbate after time *t* (mg/L) and *k*_1_ is first order rate constant. A straight line of the ln*C*_t_ versus *t* plot suggests the validity of this kinetic model. Figure 3 shows that the adsorption of Rh-B on UBTL follows simple first-order kinetic equation only at lower concentrations.

### Second-order kinetics

**Figure 4:**
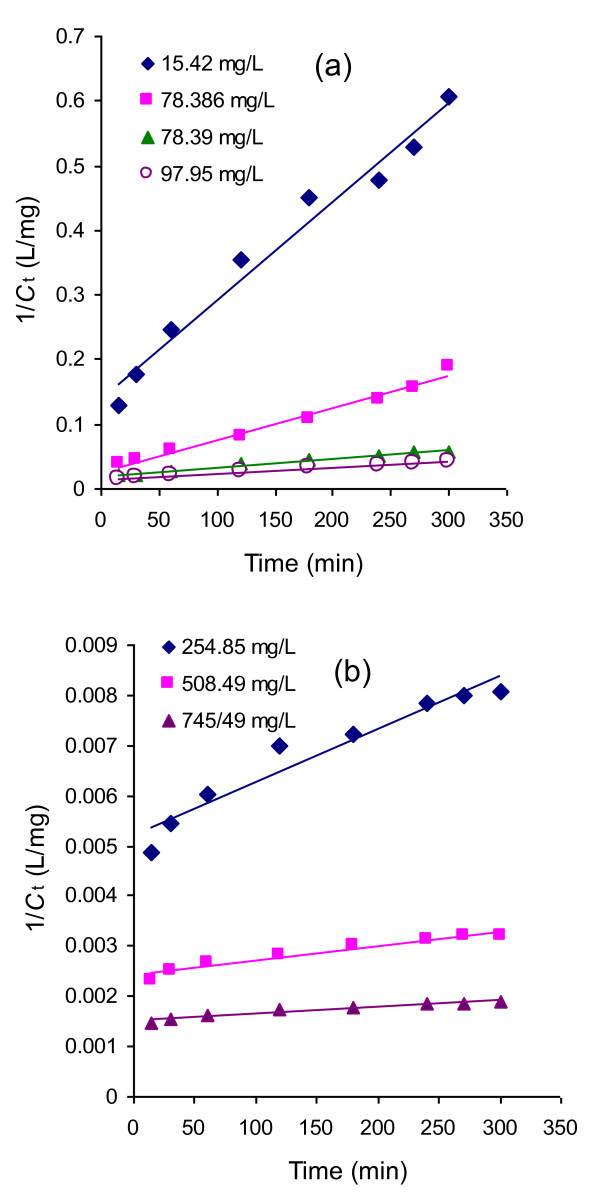
(a and b): Second-order kinetic plots of the adsorption of Rh-B on UBTL at pH = 2.0 and 33 ± 0.5°C for different initial concentrations.

To verify the dependency of the concentration of Rh-B and UBTL on the adsorption process, the integrated form of second-order rate equation (2) [15] was applied to our experimental results.

(2) $$\frac{1}{C_{\text{t}}}=k_{2}t+\frac{1}{C_{\text{o}}}$$

where *C*_o_ and *C*_t_ are the concentration (mg/L) of Rh-B at initial stage and after time *t*, respectively. Second-order rate constant is presented by *k*_2_. Figure 4 shows the applicability of the second order rate equation by plotting 1/*C*_t_ as a function of time *t*. The figure shows that the adsorption of Rh-B on UBTL follows second-order kinetics satisfactorily only at high concentrations of Rh-B.

### Pseudo-second-order kinetics

**Figure 5:**
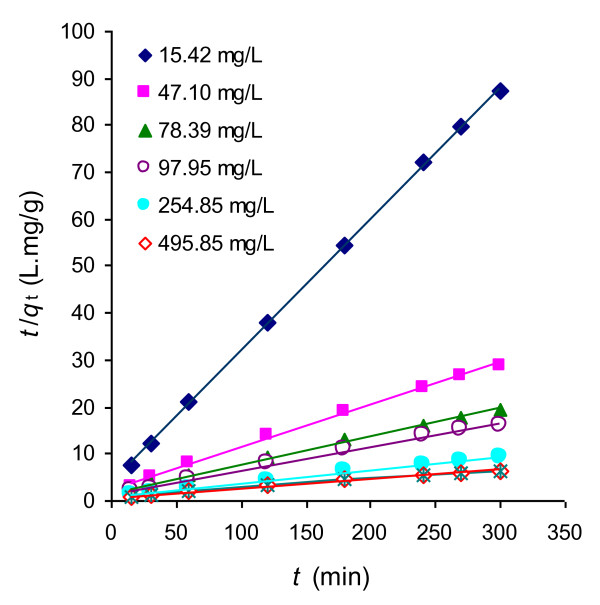
Pseudo-second order kinetic plots of the adsorption of Rh-B on UBTL at pH = 2.0 and 33 ± 0.5°C for different initial concentrations.

**Figure 6:**
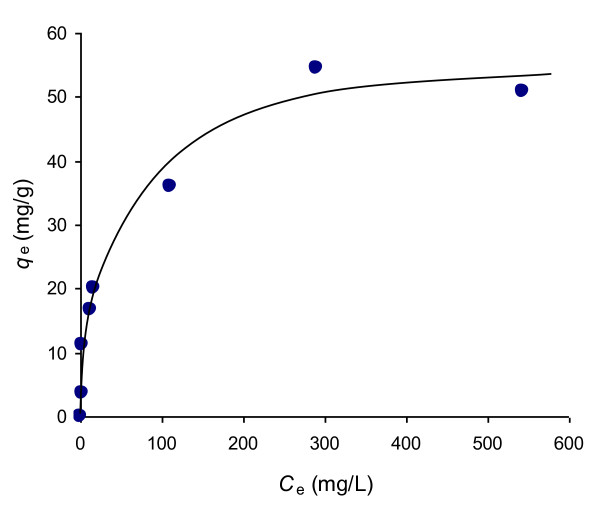
Adsorption isotherm of Rh-B on UBTL at pH = 2.0 and 33 ± 0.5°C determined from pseudo-second order kinetic plots of different initial concentrations.

**Figure 7:**
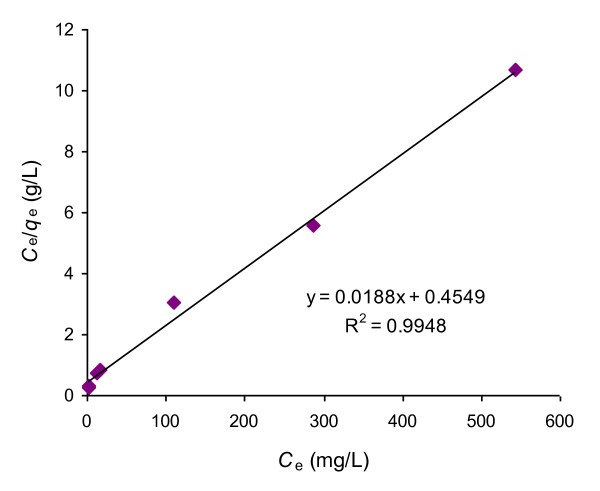
Langmuir isotherm for the adsorption of Rh-B on UBTL at pH = 2.0 and 33 ± 0.5°C.

Ho and McKay’s [16] pseudo-second order rate equation was used to express the adsorption of Rh-B on UBTL at different initial concentrations. The linearized from of Ho and McKay’s pseudo-second-order rate equation is shown in equation (3):

(3) $$\frac{t}{q_{\text{t}}}=\frac{1}{kq_{\text{e}}^{2}}+\frac{1}{q_{\text{e}}}t$$

where *q*_t_ is the amount adsorbed at time t (mg/g), *q*_e_ is the equlibrium amount adsorbed (mg/g) and *k* is the pseudo-second-order rate constant (g/mg·min). The verification of pseudo second-order kinetics for the syetem is presented in Figure 5 by plotting *t*/*q*_t_ vs. *t*. This figure shows the satisfactory fitness of each data to the straight line at all concentrations of Rh-B. From the straight lines, the equilibrium amount adsorbed for different initial concentrations of Rh-B were calculated and the variation of equilibrium amount adsorbed with equilibrium concentration is shown in Figure 6 as a representation of adsorption isotherm. The nature of isotherm was characterized by the plot of *C*_e_/*q*_e_ vs *C*_e_ as a representation of Langmuir equation (4):

(4) $$\frac{C_{\text{e}}}{q_{\text{e}}}=\frac{1}{q_{\text{m}}b}+\frac{C_{\text{e}}}{q_{\text{m}}}$$

where, *q*_e_ is the amount adsorbed per unit mass of adsorbent (mg/g), *C*_e_ is the equilibrium concentration of the adsorbate (mg/L), *q*_m_ is the monolayer adsorption capacity (mg/g) and *b* is the adsorption desorption equilibrium constant (L/mg). Figure 7 shows that the adsorption of Rh-B on UBTL at pH = 2.0. follows Langmuir equation and the calculated value of monolayer adsorption capacity, *q*_m_ = 53.2 mg/g.

### Effect of temperature

**Figure 8:**
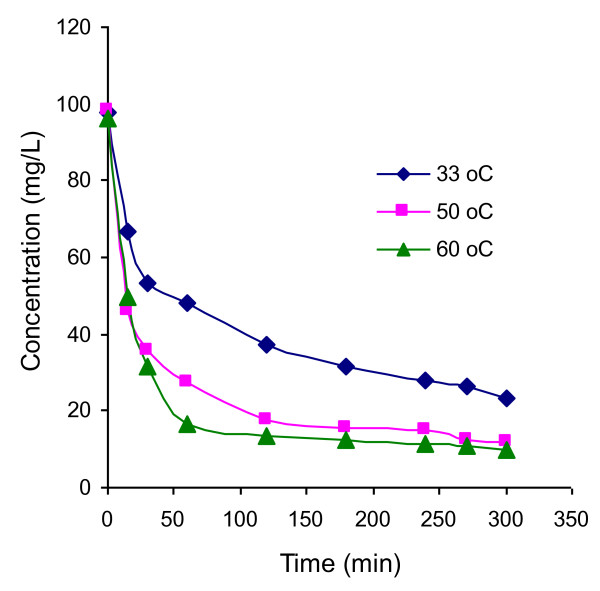
Variation of concentration with time during the adsorption of Rh-B on UBTL at pH = 2.0 and concentration 100 mg/L for different temperatures.

The effect of temperature on the adsorption kinetics was investigated by performing adsorption kinetic experiments at different temperatures. Figure 8 shows the variations concentration of Rh-B with time at different temperatures for the same initial concentration of Rh-B and the dose of UBTL. Pseudo- second-order kinetic equation was also applicable for the system at different temperatures and the respective parameters were determined. The equilibrium amount adsorbed was found to increase with temperature.

### Adsorption enthalpy

**Figure 9:**
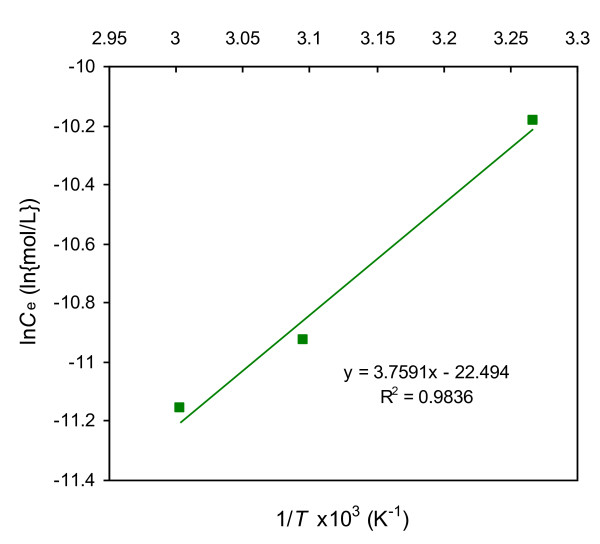
**A plot of ln *C***_**e **_**vs. 1/*T*** **× 10**^**3 **^**for adsorption of Rh-B on UBTL at pH = 2.0.**

The enthalpy of adsorption of Rh-B on UBTL at pH = 2.0 was also determined from the findings of the effect of temperature on adsorption and using equation (5) [17].

(5) $${\left[\frac{d\ln C_{\text{e}}}{\text{d(1/}T)}\right]}_{\text{θ}}=\frac{\Delta H_{\text{ads}}}{R}$$

where, *θ* indicates the fraction of surface coverage and Δ*H*_ads_ is the enthalpy of adsorption. For a particular amount adsorbed, the change of equilibrium concentrations with temperature has been calculated from Figure 8. The enthalpy of adsorption was determined from the plot of ln*C*_e_ vs.1/*T* as shown in Figure 9. The slope of the straight line of the plot is equivalent to the Δ*H*_ads_/*R*. The calculated value of enthalpy change, Δ*H*_ads_ = 31.22 kJ/mol at pH = 2.0, which indicated that the adsorption of Rh-B on UBTL is an endothermic process.

## Discussion

The UV-visible spectrum of a compound depends on its nature and structure. Since the basic dye Rh-B in aqueous solution exists as an ionic species, the absorption spectrum should be influenced by the solution pH. Thus the absorption spectrum as well as the calibration curve was constructed for analysis of Rh-B solution at a fixed pH of 2.0. All sample solutions during the adsorption study were analyzed by measuring the absorbance of solution at pH = 2.0.

The physico-chemical properties of the adsorbent were measured by various standard procedures and its high adsorption capacity was found [10]. The SEM microgram in Figure 1 shows the heterogenic nature of UBTL surface. The zero point charge pH of UBTL (pH_zpc_ = 4.2) indicated its adsorptive characteristics to negative species at low pH [10].

The first-order, second-order and pseudo-second-order kinetic constants in the linear equations were calculated from the slope and intercept of the plot between ln*C*_t_*vs*. *t*, 1/*C*_t_*vs t* and *t*/*q*_t_*vs*. *t*, respectively. A comparison of the regragration factor of linear plots for different kinetic equations are shown in Table 1. It can be concluded that the best fitted equation for the adsorption of Rh-B on UBTL is pseudo-second-order. Several studies have been reported about the fitness of pseudo-second-order kinetics for the adsorption using heterogeneous surfaces like tea leaves [18], moss peat [16], clay-wood sawdust [19], rice husk [20] and peanut hull carbon [21].

**Table 1:** A comparison of the regragration factor of the linear plots of different kinetic equations for the adsorption of Rh-B on UBTL at pH = 2.0

**Initial concentration (mg/L)**	***R***^**2**^		
	**First-order**	**Second-order**	**Pseudo-second-order**
	**kinetic**	**kinetic**	**kinetic**
15.42	0.9071	0.9755	0.9997
47.097	0.9864	0.9817	0.9973
78.386	0.9557	0.9926	0.9982
97.946	0.9617	0.9881	0.9962
254.852	0.9003	0.9336	0.9982
495.493	0.9206	0.9436	0.9952
745.489	0.9361	0.9501	0.9938

The well expressed pseudo-second-order kinetics suggested that the overall rate of Rh-B adsorption process is chemical reaction controlled [16]. The variation of equilibrium amount adsorbed with equilibrium concentration, calculated from pseudo-second-order kinetics and presented in Figure 6, indicated that the equilibrium amount adsorbed increased with the initial concentration of adsorbate as well as equilibrium concentration and became a steady value after reaching the maximum adsorption capacity. This is due to increase in driving force of the concentration gradient with increasing initial dye concentration. This observation indicates the validity of Langmuir equation to the system whatever the UBTL surface is heterogeneous. Such type of monolayer adsorption of Cr(VI) on UBTL previously was confirmed by Scanning Electron Microscopic study [10].

The amount adsorbed increased with increase in temperature suggesting the temperature dependent chemical interaction occurred between Rh-B molecules and UBTL. As the temperature increases the activation energy of interacting species also increases [18,22] due to the increased velocity of the solute [23] and the adsorption rate also increases. Again the positive value of enthalpy of adsorption suggested that the adsorption of Rh-B on UBTL at pH = 2.0 is endothermic which might be due to the fragmentation of Rh-B on UBTL surface at high temperatures.

## Competing interests

The authors declare that they have no competing interests.

## Authors’ contributions

Both MAH and MSA 1) have made substantial contributions to conception and design, or acquisition of data, or analysis and interpretation of data; 2) have been involved in drafting the manuscript or revising it critically; and 3) have given final approval of the version to be published. All authors read and approved the final manuscript.

## Authors’ information

MAH received B. Sc. Hons. in Chemistry (Dhaka University), M. Sc. in Physical Chemistry (Dhaka University), MS in Material Engineering (Kanazawa University, Japan) and Ph. D. in Environmental Science and Technology (Kanazawa University, Japan). Currently Associate Professor, Physical Chemistry Section, Department of Chemistry, University of Dhaka, Bangladesh. MSA received B. Sc. Hons. in Chemistry (Dhaka University). Currently M.S. student in Physical Chemistry, Dhaka University, Bangladesh.
